# Evaluating the Safety and Efficacy of a Highly Viscous 33-mg/mL Hyaluronic Acid Volumizing Filler in the Treatment of Facial Wrinkles: An Open-Labeled, Clinical Trials

**DOI:** 10.31661/gmj.v8i0.1148

**Published:** 2019-01-01

**Authors:** Elnaz Razavian, Setareh Tehrani

**Affiliations:** ^1^Tehran Medical Sciences Branch, Islamic Azad University, Tehran, Iran; ^2^Department of Dermatology, Tehran Medical Sciences Branch, Islamic Azad University, Tehran, Iran

**Keywords:** Hyaluronic Acid, Wrinkles, Aging Face, Dermal Filler

## Abstract

**Background:**

The 33-mg/mL hyaluronic acid (HA) formulation is a highly concentrated, cross-linked, cohesive, smooth, and completely reversible volumizing filler approved by Conformité Européene. For the first time, we aimed to evaluate the long-term efficacy and safety of the 33- mg/mL HA filler for soft tissue augmentation in the treatment of facial wrinkles.

**Materials and Methods:**

After optimal wrinkle correction was achieved in the patients undergoing treatment by injecting the 33-mg/mL HA filler at the injection site plus one touch-up at a 2-week interval, the safety and efficacy of the filler were assessed on the 5-point Facial Volume Loss Scale through the 1-year study period. Patients were evaluated daily for 14 days and after 6 and 12 months post-treatment.

**Results:**

A total of 86 subjects were treated. The mean wrinkle scores of the patients were 3.95+0.79 (range of 3-5) before treatment, 2.3+0.94 (range 1-5) six months after treatment, and 2.93+1.29 (range of 1-5) one year after treatment. Clinically significant mean wrinkle correction (P=0.001) was still evident at>12 months of treatment through 33-mg/ mL HA formulation. A clinically significant correction at>12 months after treatment was maintained by 79% of patients. Nodule formation and swelling were more frequent when the 33- mg/mL HA filler was used compared with the use of less concentrated HA fillers. One patient developed angioedema-like swelling and induration last few months.

**Conclusion:**

The 33-mg/ mL HA filler can provide long-term correction lasting for one year or more. Adverse effects, especially swelling and nodule formation were more common in this filler compared with less concentrated HA fillers. The side effects were correlated with the volume of the injected filler. We recommend using this concentration with low volume or combining high volume with lower concentration.

## Introduction

Facial aging is caused by intrinsic and extrinsic factors such as smoking, genetics, muscle activity, and sun exposure [1-4]. Facial aging changes appear as a result of the loss of elastin, loss of bone mass, atrophy of soft tissue, as well as altered collagen production [5].These changes are associated with skin laxity and volumetric loss. Multiple treatment options are available for these skin changes. One of them is treatment by dermal fillers. Dermal fillers help retrieve facial volume and reduce facial rhytides without any surgical approach. The Food and Drug Administration (FDA) approved more than 13 injectable fillers between 2000 and 2011. Showing a 1-year increase of 8.6%, United States reported 995,000 soft-tissue filler procedures in 2013. A glycosaminoglycan disaccharide named hyaluronic acid (HA), with a half-life of 3 days or less is naturally found in the human body, especially in skin [6].HA fillers play an integral part in the correction of changes associated with aging [7]. They are categorized as resorbable (such as HA) and permanent or non-resorbable (such as silicone) [8]. Every patient showing signs of skin aging wants ideal fillers that are easily injectable, yield reproducible results, and have a long-lasting effect. HA forms a major part of the extracellular matrix of the dermis. An average human body of 60 kg contains about 12 g of HA. This HA provides a space for the movement of cells as well as diffuses hormones and nutrients. It also stimulates the production of collagen besides fibroblast proliferation and migration. It regulates cell-to-cell and cell-to-matrix interactions by stimulating the cell membrane receptor CD44, thereby leading to regulated cell proliferation and motility [9].The cross-linking of HA with low molecular weight results in significantly increased viscosity [10]. The cross-linking agent 1,4-butanediol diglycidyl ether (BDDE) is used to cross-link the monophasic HA dermal fillers with the cross-linking grade between 1% and 20%. The concentration of the HA filler is between 13.5 and 25 mg/mL (such as Juvéderm and Teosyal). Biphasic HA formulation consists of HA particles in suspension with a non cross-linked HA-diluted solution. The commonly used cross-linking agents are divinyl sulfone (DVS), diepoxy octane, and BDDE. The concentration of the HA fillers such as Matridur, Restylane, Perlane, Sub Q, and Puragen is between 20 and 25 mg/m [11].The product we used in this study was Variofil, a smooth, highly cohesive, viscous HA filler with the concentration of 33 mg/mL. It is prepared from a non cross-linked HA by cross-linking it with DVS. Its cross-linking grade is 70% due to an extended cross-linking process [11]. This filler is produced by a German company, ADODERM GmbH, and approved by Conformité Européene (CE). It has been used mainly for body contour. We designed this study to evaluate the long-term effectiveness and safety of 33-mg/mL HA for facial wrinkle treatment. This study aimed to determine the use of highly concentrated HA fillers for the treatment of facial volume loss, having long-term sustainability with less frequency of injection and less cost for the patients, and also to evaluate their adverse effects because of high concentration.

## Materials and Methods

### Participants

**Figure 1:**
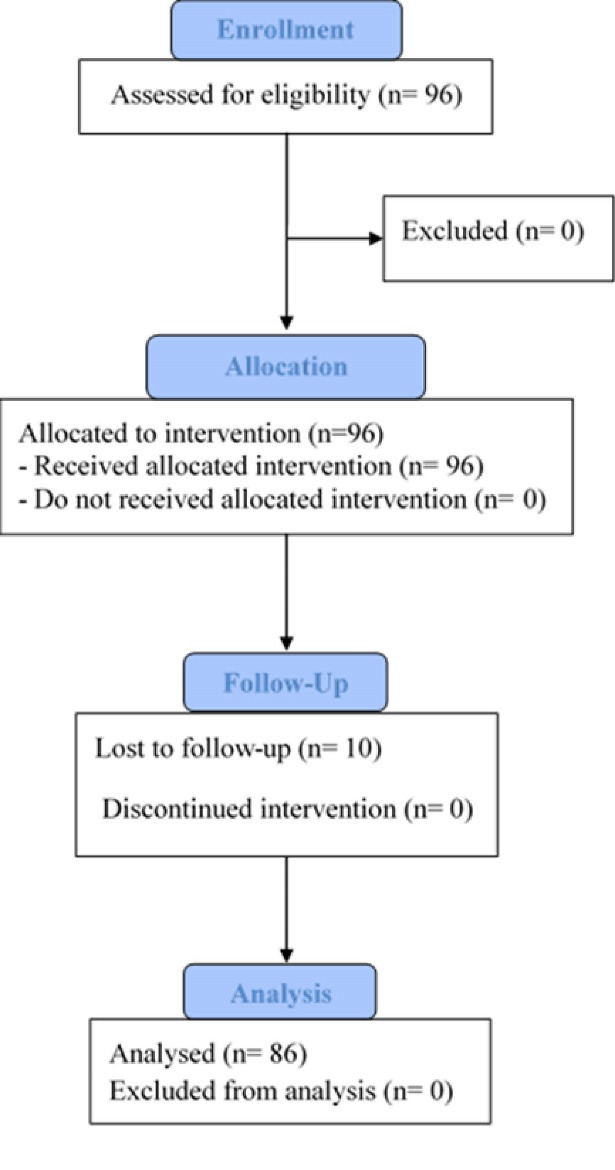
Flowchart of study

This open-label, the non-randomized clinical study was performed on 96 patients aged 40-60 years who attended our dermatology clinic during 2016-2017 at Tehran. Patients with facial volume loss scores of 3 to 5 based on the 5-point Facial Volume Loss Scale were included in this study. None had any exclusion criteria which include a history of receiving any treatment (include permanent, semi-permanent filler, implant) within the past 12 months, active infection near the site of injection, known allergy or hypersensitivity to HA, tendency to develop hypertrophic scars, autoimmune disease, active skin disease, intake vitamin E and aspirin two weeks before injection, pregnancy, and lactation. Figure-1 shows the flowchart of study. All 96 patients received the allocated intervention, and 86 participants completed all study procedures and follow-up. No participants were excluded from analyses.

### Trial Design

**Figure 2:**
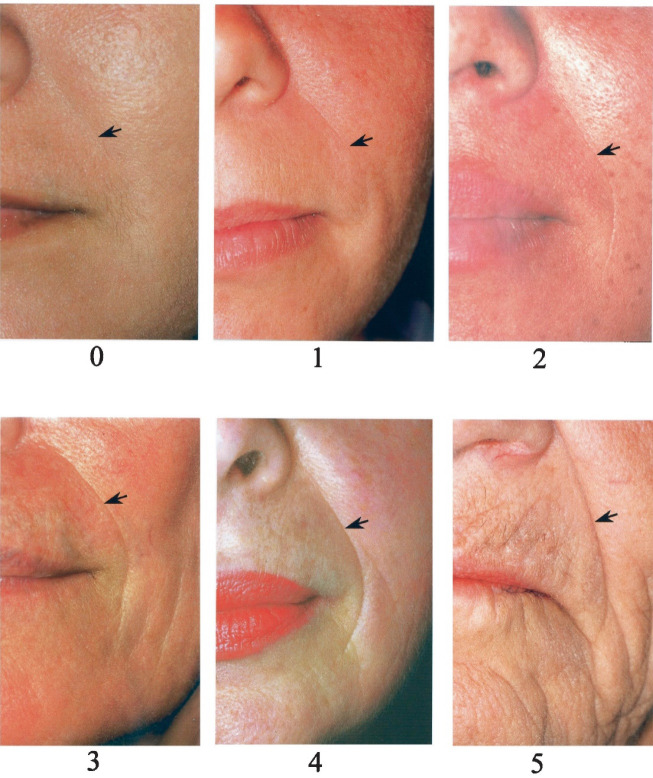
Pattern to determine the score of facial wrinkles [12].

At the day of administering the injection, the facial volume loss scores of the patients on the 5-point Facial Volume Loss Scale were determined as follows: 0=no wrinkle, 1=just perceptible wrinkles, 2=shallow wrinkles, 3=moderately deep wrinkles, 4=deep wrinkles with well-defined edges, and 5=very deep wrinkles with the redundant fold (Figure-2) [12]. The patient’s age, sex, injection site, and volume per site were recorded. We injected a 33-mg/mL cohesive filler with either a cannula or needle subcuticularly. Topical anesthesia (include 23% lidocaine and 3.5% tetracaine HCl) was used for most of the patients. The injection score after treatment was 0 for all the patients. One touch-up at a 2-week interval was performed for having an optimal correction. If there was any visible line or wrinkle mentioned, we injected more filler on a touch-up day. The patients were evaluated daily for 14 days and after 6 and 12 months to determine the safety and longevity of the filler. The facial volume loss scores were recorded on each visit. Photographs were taken pre- and post-treatment at both visits. Site, severity, and duration of any adverse events following intervention were recorded and categorized into acute, intermediate, and long-term side effects. Percentage, mean, and ranges were used to report descriptive analysis.

### Ethical Issue

The patients were informed about the objectives of the research, as well as the possible complications before the beginning of the treatment. Written consent was obtained from each participant after getting mentioned descriptions before starting the process. The Ethical Committee of Tehran Medical Sciences Branch, Islamic Azad University has approved this study (IR.IAU.TMU.REC.1396.121), and it also registered in the Iranian Registry of Clinical Trials (RCT code: IRCT20180228038905N1).

### Statistical Analysis

All data were analyzed by using the IBM SPSS Statistics for Windows, version 22 (IBM Corp., Armonk, N.Y., USA). via paired sample t-test and analysis of variance (ANOVA). The statistical significance level was set at P≤0.05.

## Results

**Figure 3:**
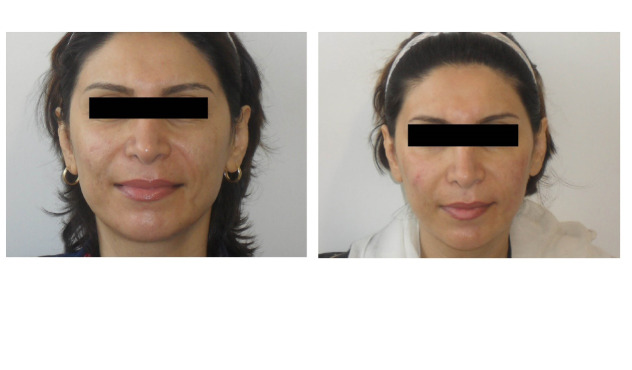
Injection of 1.5-mg/mL filler in the NLF. (**A**) Before injection and (**B**) after 12 months

**Figure 4:**
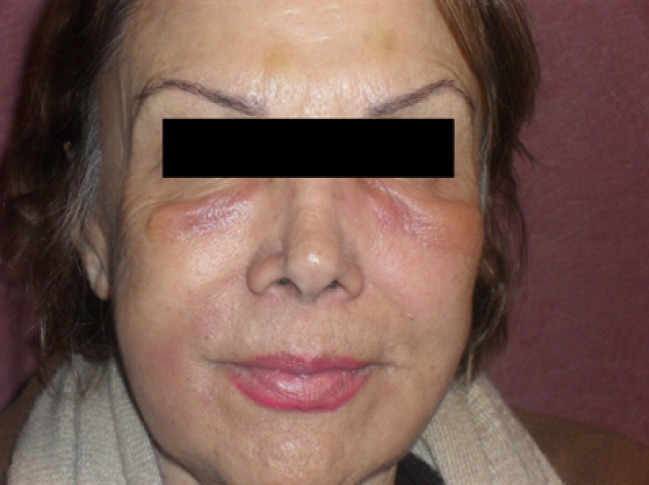
Angioedema-like swelling with the injection of 33-mg/mL filler for the augmentation of malar, NLF, and marionette lines

In the present study, 71 females and 15 males were complete follow-up and included in the final analysis. The mean age of patients was 53 ±6.08. Thirteen patients (15%) were deemed to require touch-up treatment, with a mean volume of 0.51 ± 1.09 mL. The most frequent injected site was the nasolabial fold (NLF) area; it comprised 60% of the total injection sites. The mean injection volume for this site was 1.05 ± 0.81 mL per site. The next most commonly injected site (21% of procedures) was the marionette line with a mean injection volume of 0.81 ± 0.21 mL. The malar area included 15% of the total injection sites, and the mean injection volume for the wrinkle correction of this area was 0.7 ± 0.38 mL. The remained area accounted 4% on the injected site with the mean volume of 0.8 ml±0.1.The most common scoring grade before treatment was four (70% of patients) with a mean score of 3.95 ± 0.79 (Table-1). Six months after treatment, the most common scoring grade was two (47% of patients) with a mean score of 2.3 ± 0.94 and one year after treatment, it was three (42% of cases) with a mean score of 2.93 ± 1.29. Clinically significant mean wrinkles correction (at least 1-point improvement) was evident six months and one year after injection of 33 mg/mL HA. A total of 68 patients (79%) maintained the correction for one year (Figure-3). There was no significant correlation between the age and sex of the patients and the longevity of the filler (P>0.05). However, a significant relationship was found between the volume of the filler injected and its longevity (P=0.01). The more the volume of injection, the more the longevity. Also, there was a significant correlation between the site of injection and longevity (P=0.001). The least longevity was mentioned in NLF, whereas the most in marionette lines. As shown in Table-2, 44.18% of the patients experienced no adverse effects. The mean duration of the reaction was 4.8 days. The most frequent side effects were swelling at the site of injection (20 patients), followed by nodular lesions and indurations in 12 cases at 16 sites. In most of the patients, the nodules were resolved with the help of pressure, facial massage and intense pulsed light treatment after several weeks. In the remainder, any such issue was fully resolved several months after injection, maximum six months. One patient developed angioedema-like swelling in the face four days after injection, which further increased after ten days of injection (Figure-4). The patient was treated with a systemic steroid dexamethasone (intramuscular) and prednisolone 40 mg/d for 20 days and tapered within weeks with the help of cetirizine and antibiotics (cephalexin and tetracycline). The edema resolved completely 25 days after injection.

## Discussion

Correction of facial wrinkles is one of the most important cosmetic procedures. To the best of our knowledge, this is the first study to evaluate the use of a highly viscous 33-mg/mL HA volumizing filler for treating facial wrinkles. This trial was designed to determine the efficacy and safety of this filler in patients with facial volume loss. Treatment of patients with facial aging by using a 33-mg/mL HA filler resulted in a statistically significant improvement, lasting beyond 1 year; however, it was not permanent. Highly concentrated cross-linked HA fillers that are the safest and approved by FDA and CE may last for more than six months. The use of HA fillers such as Juvéderm Ultra and Ultra Plus (24mg/mL) resulted in clinically significant mean wrinkle correction at >9 months [13]. Restylane and Perlane (20 mg/mL) resulted in the maintenance of wrinkle correction by investigators. Elevess (28 mg/mL) was used in 60% to 80% of patients with mild and moderate folds and 40% to 60% of patients with deeper folds [14-16]. The assessments suggest that six months after the last treatment, Juvéderm Ultra, and Ultra Plus made a clinically significant improvement in the mean wrinkle severity (1.3 and 1.5 points, respectively) [17], whereas Restylane (0.9 points) or Elevees did not (0.8 points) [15, 17]. All cosmetic procedures might result in undesirable adverse effects. Therefore, it is imperative to review all known potential side effects with the patient before any procedures [18]. Some adverse reactions are localized and temporary, including pain, induration, swelling, nourishing, itching, erythema, acne form eruptions, transient lumpiness, and sterile abscess [15, 18, 19]. Few adverse reactions were reported before 2010, and most of them were considered to be related to the treatment procedure. However, recent studies have reported hypersensitivity reactions to HA and another gel component [8, 20-23]. Previous studies have mentioned that these adverse effects, especially allergic ones, may be attributable to the protein component of HA associated with the impurities of the fermentation process [24, 25]. Some adverse effects such as fever, arthritis, skin lesions, arthralgia, angioedema, skin induration, edema, nodules with or without fistulation, and pus discharge are delayed immune-mediated reactions (DIMR), which may be intermediate (1-12 months after injection) or delayed (12 months or more after injection) [19]. Studies by physicians reported different rates of injection-related reactions when using Restylane, Perlane, or Juvéderm from 13% to 34% or even to 80% [14, 26]. The overall incidence of DIMR is believed to be low. A review of 709 patients [27] treated with Hyaloform or Restylane demonstrated an incidence of DIMR of less than 0.5%. Another retrospective study of 4,320 patients treated with Restylane reported an approximately 0.6% incidence of DIMR [28]. Only one case of angioedema-like swelling of the lip was recorded [29], although previous studies have reported this as a very common phenomenon following the injection of Restylane into lips [30]. Although there is no definite treatment modality for the correction of HA filler complications, we can manage them with various available treatment modalities (such as hyaluronidase injection) to minimize patient’s morbidity [31]. In this study, adverse effects including swelling, bruising, nodules, and indurations were more common and frequent when compared with administration of less concentrated HA fillers (24% of injected sites). Most of the nodular lesions were resolved with pressure and facial massage for several weeks after injection, but six lesions including 3% of injected sides persisted several months (intermediate reaction), and three lesions, including 1.7 of sites persisted even 12 months after injection (delayed reaction).

## Conclusion

Our study shows that the 33-mg/mL HA filler could correct facial wrinkles lasting for one year or more with less frequent injection and less cost. Adverse effects, especially swelling and nodule formation were more common compared with the use of less concentrated HA fillers. However, most of them were transient and correlated with the volume of the injected filler. We recommend using this filler with the combination of high concentration and low volume or high volume with lower concentrations.

## Acknowledgment

We would like to thank the Islamic Azad University Tehran Medical Branch and ADODERM GmbH.

## Conflict of Interest

None declared.

**Table 1:** Volume Loss Ratings Based on the Wrinkle Classification Changes

	**Wrinkles score** **(%)**						**Mean±SD**	**Range**
	**0**	**1**	**2**	**3**	**4**	**5**		
**Before injection**	0	0	9%	10%	70%	11%	3.95+0.79	3-5
**6 months**	0	33%	47%	18%	2%	0	2.3 + 0.94^a^	1-5
**12 months**	0	2%	30%	42%	21%	5%	2.93+1.29^b^	1-5

^a^ P<0.01, ^b^ P<0.05 vs. baseline

**Table 2:** Adverse Effects in Studied Patients

**Adverse effects**	**N**	**%**
**Swelling**	20	23.25
**Nodules and/or indurations**	16	18.6
**Bruising**	5	5.81
**Bruising and Swelling**	3	3.5
**Persistent Nodules (>1year)**	3	3.5
**Angioedema swelling**	1	1.16
**None**	38	44.18
